# Geographic and Temporal Trends in Influenzalike Illness, Japan, 1992–1999

**DOI:** 10.3201/eid1010.040147

**Published:** 2004-10

**Authors:** Takatsugu Sakai, Hiroshi Suzuki, Asami Sasaki, Reiko Saito, Naohito Tanabe, Kiyosu Taniguchi

**Affiliations:** * Niigata University School of Medicine, Niigata, Japan;; † National Institute of Infectious Disease, Tokyo, Japan

**Keywords:** Influenza, surveillance system, GIS, kriging, pandemic, research

## Abstract

From 1992 to 1999, we analyzed >2.5 million cases of influenzalike illness (ILI). Nationwide influenza epidemics generally lasted 3–4 months in winter. Kriging analysis, which illustrates geographic movement, showed that the starting areas of peak ILI activity were mostly found in western Japan. Two spreading patterns, monotonous and multitonous, were observed. Monotonous patterns in two seasons featured peak ILI activity that covered all of Japan within 3 to 5 weeks in larger epidemics with new antigenic variants of A/H3N2. Multitonous patterns, observed in the other five seasons, featured peak ILI activity within 12 to 15 weeks in small epidemics without new variants. Applying the kriging method allowed better visualization and understanding of spatiotemporal trends in seasonal ILI activity. This method will likely be an important tool for future influenza surveillance in Japan.

## Abstract 2

Kriging analysis improved visualization and understanding of trends in seasonal ILI activity in Japan.

Influenza is a highly contagious acute respiratory disease that has caused global epidemics and pandemics. Pandemics in the 20th century have occurred at intervals of 11 to 39 years (*1**–**3*). The World Health Organization has requested each member state to produce a pandemic plan. The phasing and geographic spread of influenza pandemics have important implications for future planning, and complete global spread is now likely to occur in <6 months, as a result of increased travel and urbanization (*4*).

The National Epidemiological Surveillance of Infectious Diseases in Japan features sentinel surveillance for 27 infectious diseases, including influenza (*5**,**6*). To better understand the movement and velocity of influenza epidemic spread from 1992 to 1999 in Japan, we used a geographic information system (GIS) with generated weekly surveillance data. We focused on the kriging method to illustrate and clarify spatiotemporal relationships in epidemiologic research, e.g., for rotavirus and influenzalike illness (*7**–**9*).

## Methods

### Influenza Surveillance System in Japan

The systematic surveillance of influenza and influenzalike illness (ILI) as notifiable diseases under Infectious Disease Control Law began in 1981 in Japan. Each ILI case is defined on the basis of a sudden fever >38°C, respiratory symptoms, and myalgia. The number of patients with ILI is reported on a weekly basis from ≈2,400 sentinel pediatric and general physicians and 663 health centers throughout Japan. The number of sentinels is decided on the basis of the size of the population of the health center area where they serve: a health center with population <75,000 would have one sentinel, a population 75,000–125,000 would have two, and populations >125,000 would have three + [(population – 125,000)/100,000] sentinels (*10*). Recruitment is on a volunteer basis. Sentinels forward clinical data to ≈60 prefectural or municipal public health institutes, and data generated are electronically reported to the Infectious Disease Surveillance Center in the National Institute of Infectious Diseases (Tokyo) (*5**,**6*).

### Geographic Analysis

We analyzed surveillance data from 1992 to 1999 for 46 prefectures, excluding Okinawa Prefecture, which is approximately 800 km from the four major islands of Japan. To combine data from all prefectures and examine trends at the national level, we calculated the number of reported ILI cases per sentinel per week after adjusting the epidemic curves with a 5-week unweighted moving average (reported ILI cases per sentinel per week [RC/S/W]) as an indicator of ILI activity. This procedure smoothed the data and simplified identification of the seasonal peak (*7*). The peak week during each influenza season was defined when the greatest unweighted moving average was observed in individual prefectures. For the time scale of geographic analysis, the first week was defined when the first peak was observed in any of the prefectures during the season; subsequent weeks were then numbered accordingly.

For the spatiotemporal spread of the 1992–1999 epidemics in Japan, we used the kriging geostatistical method to estimate point values by using surrounding, known point values (*11**,**12*). The address of the prefecture government was used as the representative site of prefecture surveillance data, and unweighted moving average for each prefectural peak week was applied after adjusting the time scale. Kriging uses a weighted moving average interpolation to produce the optimal spatial-linear prediction. The estimated kriging weight matrix is a product of the inverse covariance weight matrix and the distance matrix (*11**,**12*).

To make kriging maps as contour maps showing the timing of peak ILI activity, we performed the following steps. First, we created an empiric semivariogram to examine the structure of data. The empiric semivariance is 0.5 times the difference squared, when Euclidean distance is used. Second, this semivariogram estimated the theoretical model parameters through a weighted least-squares technique. The data showed a spherical pattern. Next, the weights were determined by incorporating the spherical pattern of covariance. Finally, we estimated the values at unmeasured points and made filled-contour maps from the kriging weights for the measured values. The isobars on the contour maps represent interpolated time of peak activity distributed spatially and were placed at 1-week intervals. All procedures were carried out on ArcGIS 8.2 (ESRI, Redlands, CA) and Geostatistical Analyst (ESRI) for Windows.

### Statistical Analysis

To ascertain the relationship between epidemic scale and velocity of spread, we used three parameters. greatest number of ILI cases, increasing-to-peak period, and nationwide peak-duration. The first parameter was the greatest number of ILI cases, defined as the greatest number of RC/S/W in each prefecture The second parameter was the increasing-to-peak period, defined as the time from the week RC/S/W was >50% of peak to the week of the peak. Influenza epidemics usually show an elevated incidence of ILI before the peak and for some weeks after each epidemic. In our study, sharp increases in ILI cases were seen in the weeks before the epidemic; we focused on these weeks. The means for the two parameters across prefectures were calculated for each season. The third parameter was nationwide peak-duration, defined as the time between the first and last week that showed the greatest number of ILI cases among 46 prefectures in each season. Spearman's correlation coefficient was used to analyze the relationship between all pairs of the three indexes. All calculations were performed with Microsoft Excel 2002 (Microsoft Corp., Redmond, WA), and significance was determined at p < 0.05.

## Results

### Influenza Epidemics, 1992–1999

**Figure 1:**
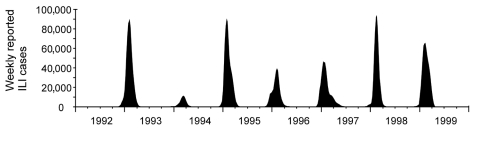
Moving averages of weekly reported influenzalike illness (ILI) cases.

We analyzed 2,586,272 ILI cases during the 7-year period from 1992 to 1999. The annual influenza season began between November and December, peaked between January and February, and returned to baseline between April and June for the study period in every year at all reporting sites (Figure 1). Seasonal peaks in ILI activity occurred annually in all prefectures. Nationwide epidemics lasted for 3–4 months, but successive or overlapping waves of infection by influenza A and B sometimes resulted in a more prolonged outbreak, as in the 1996–1997 season.

The predominant circulating strain was A/H3N2 in all seasons, except in 1996, when it was A/H1N1. Larger scale epidemics were observed in the 1992–93, 1994–95, and 1997–98 seasons, when new antigenic variants of A/H3N2 as predominant circulating subtypes were isolated, namely A/Kitakyushu/159/93, A/Wuhan/359/95, and A/Sydney/5/97, respectively. A relatively large-scale epidemic was also observed in the 1998–99 season, but the predominant circulating subtype was A/Sydney/5/97, as in the previous year. The first peak arose from one predominant viral agent and was bigger than the second peak in the 1996–97 season, with a bimodal curve.

### Geographic Analysis

**Figure 2:**
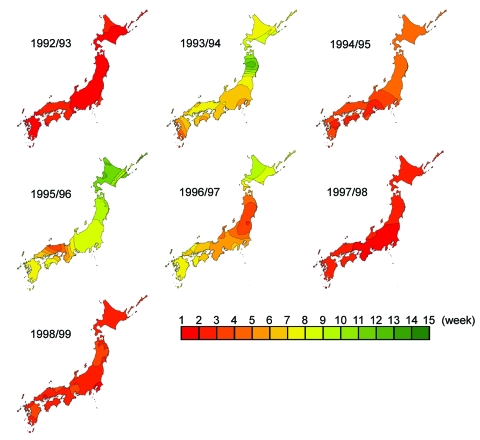
Timing of peak influenzalike illness epidemic activity by week in Japan. The isobars on the contour maps represent interpolated time of peak activity distributed spatially at 1-week intervals. The first week was defined when the peak week was observed first in any one of the prefectures in each season, and then the following weeks were numbered.

Kriging analysis clearly illustrated spatiotemporal movement of ILI epidemics in Japan (Figure 2). Seasonal ILI activity occurred in a sequential manner, and differences between seasons were easy to identify and characterize. The starting prefectures or areas of the peak ILI activity were mostly in the western part of Japan, except in the 1996–97 season. Trends did not change with the appearance of new variants. The most dramatic differences from year to year were in spreading pattern, as shown in the contour map of peak ILI activity by week. With the monotonous spreading pattern, peak ILI activity covered Japan within 3 to 5 weeks in large epidemics with new antigenic variants of A/H3N2, such as occurred in the 1992–93, 1994–95, 1997–98, and 1998–99 seasons. On the other hand, with the multitonous patterns, peak ILI activity covered Japan within 12 to 15 weeks in small epidemics without new antigenic variants of A/H3N2 in the other four seasons.

### Statistical Analysis

**Figure 3:**
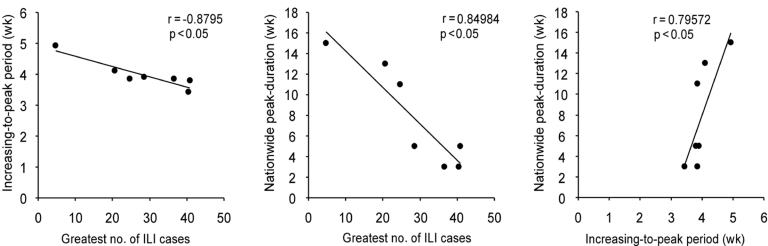
Correlation analysis among three parameters. Greatest number of cases refers to the greatest number of reported influenzalike illness (ILI) cases per sentinel per week (RC/S/W) in each prefecture in each season. The increasing-to-peak period refers to the period from the week when the number of RC/S/W reached >50% of the peak to the peak week. The means of the above two parameters were calculated by season and used for analysis. The nationwide peak-duration refers to the time between the first and last peak week observed among 46 prefectures.

During the 7-year study period, the greatest number of ILI cases, the increasing-to-peak period, and the nationwide peak duration were 4.67–40.88 ILI cases per sentinel per week, 3.43–4.83 weeks, and 3–15 weeks, respectively (Figure 3). With the larger epidemics, such as in 1992–93, 1994–95, and1997–98, and, to a lesser extent, 1998–99, the greatest number of ILI cases was >28 RC/S/W, the increasing-to-peak period was <4 weeks, and the nationwide peak duration was <5 weeks. These three parameters were interrelated (p < 0.05).

## Discussion

The size of epidemics and their relative effect reflect interplay between antigenic variation of the virus, protective immunity in the population, and relative virulence of the viruses. Kriging analysis showed several temporal and spatial patterns of influenza epidemics in Japan, which had not previously been clearly recognized.

Climate conditions, especially temperature, strongly affect influenza epidemics. Influenza in temperate areas is characterized by one annual epidemic in winter (*4**,**13**,**14*), and the influenza season occurs from November through April in Japan. The kriging map showed that the first epidemic areas with the greatest number of ILI cases in 46 prefectures were in western-central Japan during the 7-year study period, except in one season. The map showed nationwide epidemic patterns spreading in concentric circles from western-central Japan to eastern Japan. Mean temperature in winter is lower in eastern Japan than in western-central Japan, so cool temperatures are not essential to initiate epidemics.

Immunization coverage, increase in population density, and more frequent international and domestic traffic may have changed the course of epidemics and modified space-time spread (*15**–**17*). Immunization coverage is almost the same in all regions of Japan, while population density and traffic are higher in western-central Japan than in the eastern areas. Therefore, we can conclude that the last two factors may affect the nationwide spreading patterns of epidemics (*15**–**17*).

The kriging maps showed seasonal ILI activity occurring with two different patterns of peak ILI activity. Larger epidemics with new A/H3N2 variants as antigenic drift showed monotonous patterns, and these epidemics' peak ILI required only 3–5 weeks to cover the whole country. By contrast, small epidemics without new variants showed multitonous patterns, and peak ILI required 11–15 weeks to spread. A relatively large-scale epidemic with A/Sydney/5/97 was observed in the 1998–99 season, as in the previous year. The age distribution of ILI cases in the 1997–98 season was mostly <10 years of age, and in 1998 to 1999, the age distribution was mostly >15 years (*18*); these became two successive, large-scale epidemics. We conclude that kriging maps can indicate the spreading mode and velocity in conjunction with the extent of antigenic change of A/H3N2. However, the time period used for this analysis may not be representative of influenza over the long term, since we studied a period with an unusual predominance of influenza A/H3N2 viruses. Thus, we need further GIS study to know the spreading mode and velocity in conjunction with various strains.

The kriging map allowed us to better visualize and understand spatiotemporal trends in seasonal influenza activity (*8**,**9*). To confirm the GIS observations, especially the scale of epidemics and velocities, we developed three parameters: greatest number of ILI cases, increasing-to-peak period, and nationwide peak duration, which demonstrated significant interrelation (p < 0.05). We conclude that the larger the greatest number of RC/S/W found, the shorter the increasing-to-peak period and also the shorter the nationwide peak duration. As the scale of the greatest number of ILI cases obtained at the national level was connected with those from prefectural data and had an effect on the spreading mode and velocity of peak ILI activity, the greatest number of ILI cases obtained from the first prefecture in the season also is worthy of attention.

Influenza pandemics occur when a novel influenza virus emerges and most of the world's population has no immunity against it. These pandemics have been observed only with influenza A viruses, which exist in nature as a number of antigenically distinct subtypes and are due to the emergence of a novel hemagglutinin on the virus surface with or without a concomitant change in neuraminidase. In a pandemic, the number of new general practice visits for ILI can be expected to exceed 500 per 100,000 population per week; a medical practice of 10,000 patients would therefore expect to see at least 50 new patients per week (*3*). Under these conditions, our results indicate that the nationwide peak-duration might be <2 weeks. Therefore, once a pandemic begins, it will be too late to accomplish many key activities required to minimize its impact. Thus, preparatory activities must start well in advance (*19*). Stockpiling antiinfluenza drugs (*1**,**14**,**20*) seems a reasonable option until prophylactic strategies based on better vaccines can be implemented. Our results demonstrate that GIS is an effective surveillance tool to clarify the dynamics of influenza epidemics.
